# Identity of the Living and Dead

**Published:** 1910-10-08

**Authors:** 


### Medico-Legal Points.

### IDENTITY OF THE LIVING AND DEAD.

Trials where instances of mistaken identity have been the issue are so numerous and curious as to form many of the *causes célèbres* of all times, and have reflected perhaps more upon the value of human testimony than any other influence. When cases in which the identity of an individual is contested come before a court, the difference of opinion that exists will generally be of such a nature as to render the duty of the tribunal trying and difficult. This subject is calculated to excite attention, to awaken discussion, and to cause great positiveness of opinion on one or the other side. It may be stated generally that in such instances the advice of the medical man may assist in leading to the detection of falsehood and the establishment of truth. If there be anything like positive data, which cannot deceive, he can aid in their development, and they must be drawn from a source which naturally falls under his province. Plentiful cases upon record prove either how poor is the observation of the ordinary individual or how common are certain appearances which have been looked upon as more or less striking peculiarities. Many interesting questions have arisen in connection with identification, not a few of which have formed the basis of legal proceedings, and the literature of medicine is full of dramatic instances of mistaken identity. While under some circumstances persons who have had ample opportunity for observation make the gravest errors in identification, on the other hand it is sometimes the case that a momentary glance in the shortest possible association has sufficed for a perfect identification. The matter of systematic identification is largely a question of practice and skill, but in some measure depends upon the faculty of intuition, which, however, does not belong to many. It is certain that the power of unconscious observation is possessed by some individuals to a marked degree, and while those persons never forget a face, they are quite unable to explain their quickness or the means which enables them to reach a conclusion. The most astonishing examples of confident identification are found in books and the daily press, which often relate instances of individuals who were perfectly sure of the identity of another, but whose positive declarations were afterwards proved to be valueless by the appearance of the real person. Such a condition of affairs occurred in the Tichborne case; Arthur Orton was recognised, and his cause was championed not only by the mother of the real heir but by the old friends and servants of Sir Roger Tichborne. It seems almost incredible, but women have lived upon the closest intimacy with men who have turned up long after the disappearance of their lawful spouses, firmly believing them to be their long-lost husbands.

### The Case of Martin Guerre.

The most celebrated, probably, that has ever occurred, if not in Europe at least in France, is that of Martin Guerre, brought before the Parliament of Toulouse in 1560. Its incidents are so extraordinary that many have deemed it a fictitious narrative. Martin Guerre had been absent from his home for the space of eight years. An adventurer, named Arnauld Dutille, who resembled him, formed the design of taking his place, and actually succeeded so far as to be received by the wife of Martin as her husband, and to take possession of his property. Children were born to this union, and he lived three years in the family with four sisters and two brothers-in-law of Martin without their suspecting his identity. It became, however, a subject of dispute. Several hundred witnesses were examined, and of these thirty or forty swore that he was the real Martin Guerre, nearly the same number that he was Arnauld Dutille, while others deposed that the resemblance between the two men was so great that they could not decide whether the prisoner was an impostor or not. The perplexity of the judges on this occasion was very great; but in spite of many things that weakened his cause they were on the point of deciding in favour of Arnauld when the true Martin disclosed the deception. Even when confronted the impudence and effrontery of Dutille were such as to lead many to doubt, until the brother and sisters of the absent person fully recognised him.

An examination of the cases that have occurred leads to the conclusion that considerable importance should be attached to physical signs. The recollection of individuals may be weakened, and even the physiognomy of the persons in question may be altered, while marks will remain which are not to be effaced. It is on such that reliance should principally be placed.

### The Identity of the Living.

The personal identity of the living is a more frequent subject of discussion than that of the dead, constituting, as it often does, an essential connecting link in a criminal trial, and the entire subject-matter of dispute in a civil cause. Thus a man who has committed a crime leaves the country and afterwards returns. The authorities charge him with the offence, to which he pleads mistaken identity. Or again, a person, after many years' absence, returns home and claims as rightful heir title and property unrecognised by the home relatives; they dispute the relationship and kinship of the claimant. As a rule in such cases the decision of the Court turns more on general than on scientific evidence; nevertheless scientific evidence is frequently required, and oftentimes involves the most subtle of subtle scientific questions. Thus the precise nature of various corporeal defects—or the cause of sundry marks or cicatrices found on the body—or the question how far the disappearance of cicatrices known to have existed on the real person may be capable of scientific explanation, may constitute the very gist of a case.

The medical expert further will be expected to be familiar with the various scientific investigations and inquiries that have been instituted from time to time respecting natural and unnatural growths, and with the recorded observations relating to the effects of age, accident, climate, occupation, and so forth, on individuals at different intervals of time and at different periods of life. These details it will be his duty, as a medical jurist, to apply to the special case under inquiry.

### Facial and Other Characteristics.

During life the general expression of the face can be so readily altered by voluntary power that mistakes can be easily made. The notorious Charles Peace was so clever at disguising his features by voluntary movements that he was frequently able to converse without discovery with detectives who knew him. After death such voluntary alteration is, of course, impossible; but death so speedily alters expression that too much reliance must not be placed upon this mode of identification. Photography, again, is notoriously an unreliable method of identification unless minute details are considered. The details of features are, however, more enduring and more satisfactory as evidence of identity.

Cases of identity are frequently complicated by the fact that the great majority of people are untrained in minute observation. Thus one part of a family will describe an absent relative as having very dark hair, whilst another part, with equally good faith, will assert that when last seen he had light hair. Even photographs help but little in this respect, complexion, general appearance, depth of tint, and so forth depending far more on such details as the after touching-up of the negative, the time of its exposure, and the ease with which it prints than they do on the face and complexion of the sitter. Further, the recognition of a well-known friend with whom we have had daily intercourse by a carte-de-visite taken many years previously is often far from easy. In all cases where photographs are required in a court of law the negatives themselves should, if possible, be called for and produced. The tricks that a skilful photographer and toucher-up can play with a negative render prints comparatively valueless as evidence.

### Natural and Artificial Change.

In investigating the history and condition of the person whose identity is suspected it is of the utmost importance that the examiner should give weight to two kinds of influences that may effect an alteration, viz.: (1) Those in which the changes are due to age, disease, and natural or accidental alteration; (2) those in which the alterations are wilfully produced. To the former belong the organic and facial expression changes due to insanity, to trophic changes, to the loss of teeth or hair, or through cutaneous disease with pitting or other lesions. To this class belong the existence of accidentally produced cicatrices, the loss of limbs, deformities, etc., and the appearances due to manual or other labour. In the second class we find changes voluntarily wrought which are sometimes wilfully brought about for a purpose, or occasionally exist as evidences of former vanity. In this group occur cases where it is required to determine whether the hair has been dyed, whether abrasions, wounds, or burns have been made for a purpose, and whether tattoo or other marks have been removed, or, on the other hand, executed with the intention of counterfeiting the marks upon the body of some person who has disappeared or died, for the purpose of perpetrating a fraud. It is often a difficult matter to determine the identity by the colour and condition of the hair. Criminals and others, for the purpose of disguise, have by means of dyes wrought a chance in appearance which has been more or less effectual.

### Identity of Mutilated Remains.

As a rule science in its most elaborate refinements fails to dispose entirely of a dead body. A dead body partially putrefied may be found mutilated, and parts of it may be discovered in localities distant from each other. There is less difficulty here in making out identity than where only bones are discovered; for it is by no means easy to say whether certain bones belonged to the same skeleton or not. So long as the soft parts are attached to them there will be no difficulty in forming an opinion. Those who commit murder and thus dispose of a body believe that identity must be entirely destroyed if they only deposit the parts in remote places. In this respect, however, they are generally deceived, for satisfactory evidence may still be forthcoming. In the case of the woman Brown, murdered by Greenacre in 1837, the head, trunk, and limbs were scattered in widely distant parts of London. The limbs were not found until six weeks after the trunk, and then at a considerable distance and under very different circumstances.

### Bones.

The greatest ignorance prevails among the public on the subject of human and animal bones. It is therefore very important to entrust the examination of bones, in all judicial inquiries, to well-educated medical men. The lower classes of society are ever ready to suspect murder when bones are exhumed; and it is not always easy to satisfy them that the bones exhumed could not have belonged to a human being. Observations carefully made on the state of the jaws and teeth may have an important bearing in cases of disputed identity. In questions of identity, more especially in criminal trials, few things hold so important a place as, or involve investigations of greater nicety than, determining the precise nature of various spots or stains found on fabrics, instruments, etc.

One of the first questions asked on the disinterment of bones relates to the length of time during which they may have remained buried in the ground. The period at which the bones begin to undergo decomposition will depend upon that at which the soft parts have entirely disappeared. The common opinion is that the soft parts are destroyed in ordinary graves in about ten years. The changes in the bones are observed to commence by the loss of animal matter, so that they become lighter; externally they acquire a dark incrustation when in contact with the earth. This dark incrustation is sometimes confined to the surface, but in some very ancient bones the osseous shell is of a dark brown colour throughout, like old oak. The animal matter is never entirely lost; it exists in bones which have been buried for many centuries, and may be made evident by digesting them in hydrochloric acid.

